# Two-Phase Robust Target Localization in Ocean Sensor Networks Using Received Signal Strength Measurements

**DOI:** 10.3390/s21051724

**Published:** 2021-03-02

**Authors:** Yuanyuan Zhang, Huafeng Wu, Xiaojun Mei, Jiangfeng Xian, Weijun Wang, Qiannan Zhang, Linian Liang

**Affiliations:** Merchant Marine College, Shanghai Maritime University, Shanghai 201306, China; zhangyuanyuan@stu.shmtu.edu.cn (Y.Z.); meixiaojun19@stu.shmtu.edu.cn (X.M.); 201530110005@stu.shmtu.edu.cn (J.X.); 201840110003@stu.shmtu.edu.cn (W.W.); zhangqiannan02@stu.shmtu.edu.cn (Q.Z.); lianglinian@stu.shmtu.edu.cn (L.L.)

**Keywords:** received signal strength (RSS), target localization, ocean sensor networks (OSNs), majorization-minimization tactic (MMT), Cramer-Rao lower bound (CRLB)

## Abstract

Target localization plays a vital role in ocean sensor networks (OSNs), in which accurate position information is not only a critical need of ocean observation but a necessary condition for the implementation of ocean engineering. Compared with other range-based localization technologies in OSNs, the received signal strength (RSS)-based localization technique has attracted widespread attention due to its low cost and synchronization-free nature. However, maintaining relatively good accuracy in an environment as dynamic and complex as the ocean remains challenging. One of the most damaging factors that degrade the localization accuracy is the uncertainty in transmission power. Besides the equipment loss, the uncertain factors in the fickle ocean environment may result in a significant deviation between the standard rated transmission power and the usable transmission power. The difference between the rated and actual transmission power would lead to an extra error when it comes to the localization in OSNs. In this case, a method that can locate the target without needing prior knowledge of the transmission power is proposed. The method relies on a two-phase procedure in which the location information and the transmission power are jointly estimated. First, the original nonconvex localization problem is transformed into an alternating non-negativity-constrained least square framework with the unknown transmission power (UT-ANLS). Under this framework, a two-stage optimization method based on interior point method (IPM) and majorization-minimization tactic (MMT) is proposed to search for the optimal solution. In the first stage, the barrier function method is used to limit the optimization scope to find an approximate solution to the problem. However, it is infeasible to approach the constraint boundary due to its intrinsic error. Then, in the second stage, the original objective is converted into a surrogate function consisting of a convex quadratic and concave term. The solution obtained by IPM is considered the initial guess of MMT to jointly estimate both the location and transmission power in the iteration. In addition, in order to evaluate the performance of IPM-MM, the Cramer Rao lower bound (CRLB) is derived. Numerical simulation results demonstrate that IPM-MM achieves better performance than the others in different scenarios.

## 1. Introduction

**Figure 1:**
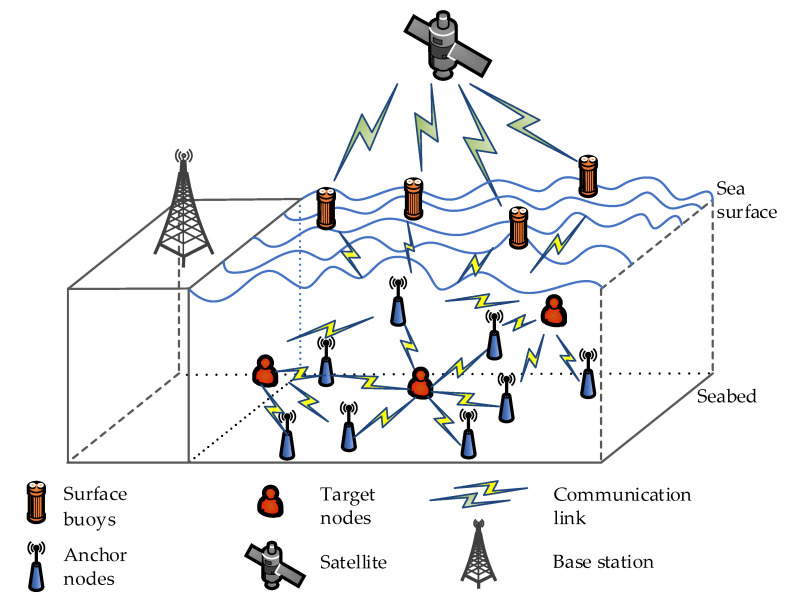
System model for the ocean sensor networks (OSNs).

In recent years, wireless sensor networks (WSNs) have been widely used in many fields [1,2,3], such as data collection, environmental monitoring, target localization and tracking, and traffic control. One of the critical applications is the use of ocean sensor networks (OSNs) to locate ocean targets [4]. In order to achieve effective and accurate positioning, some sensor nodes that are sensitive to location indicators are usually deployed on the ocean surface or underwater, including anchor nodes and unknown nodes. It deserves to be noted that the location of some anchor nodes is fixed and pre-known, and some are mobile and equipped with the localization hardware that can provide location information. The goal is to make full use of the anchor node information to locate the target node. In this paper, buoy nodes on the ocean surface, the anchor nodes underwater with the known position, and unknown target nodes are considered in OSNs where they can communicate using the acoustic signal [5,6]. The whole system model for OSNs is depicted in Figure 1.

It is well known that, on account of the dynamics and complicated communication channels in the ocean environment, underwater localization is more complicated and challenging than ground positioning [7]. Different from terrestrial radio-frequency wireless communication, the use of acoustic signal seems to be a better way in OSNs [8]. Unfortunately, it is difficult to estimate a target with the acoustic signal due to various adverse factors, including the dynamics of the environment, multipath and absorption loss of the signal, and noise interference [7,9,10]. In this context, how to locate a target in such a complex ocean environment is a challenge. 

In terms of localization methods in OSNs, it mainly includes cooperative localization (CL) scheme and non-cooperative localization (NL) scheme. The unknown target nodes can communicate with anchor nodes and other unknown nodes within a certain communication range for the former, whereas the latter cannot. Frankly speaking, the CL scheme consumes more energy and computational time, together with a higher complexity compared to NL scheme [11]. In this case, we concentrate on the NL scheme in OSNs. Among the techniques in terms of localization in NL scheme, received signal strength (RSS)-based method has gained the upsurge of interest by researchers with its low complexity, easy implementation, cost-effective, and synchronization-free, compared with time of arrival (TOA), time difference of arrival (TDOA), angle of arrival (AOA)-based techniques, and their variants [12,13,14,15].

In what concerns the RSS-based technique, various approaches have been developed to solve the localization problem for terrestrial wireless sensor networks. In [16], the authors proposed a novel RSS-based indoor localization method using belief function theory, in which they modeled RSS by non-Gaussian probability density functions and put forward decision-making modes to improve the localization accuracy. In [17], the authors formulated the localization problem into a nonlinear weighted least squares (WLS) problem, where the unscented transformation and the bisection method were adopted to estimate the transmit power and the target location jointly. In [18], a suboptimal estimator has been derived from dealing with the localization problem. Unfortunately, the suboptimal estimator seems to perform poorly under the visible bias and variance in a noisy environment. In this context, considerable efforts have been made to improve performance [19,20,21,22,23]. The authors in [20] have employed a hybrid model that combined the distance and angle measurements to reduce the estimation error. Nevertheless, the hybrid information extraction may increase the network complexity, albeit the localization accuracy has been improved. Chan et al. in [22] have designed a WLS estimator to solve the 3-D AoA/RSS Difference (RSSD) non-cooperative localization problem in a relatively small-scale WSN with low noise power. In addition, to multiple-target localization in 3-D WSNs, a linear quadratic estimator was presented in [23], which integrated RSS and AOA measurements and utilized spherical representation of the considered problem to acquire a closed solution.

However, different from the terrestrial WSNs, it remains challenging to locate targets in OSNs using RSS-based methods due to the dual factors of path loss and absorption loss for the underwater acoustic signals. The dual factors may negatively impact localization in OSNs and limit the capability of the RSS-based methods. In this case, substantial efforts have been made to improve the localization performance using RSS-based methods in OSNs [24,25,26,27,28]. In [24], the maximum likelihood (ML) problem regarding the RSS-based underwater target localization was discussed, and a series of methods were derived using the *l*_1_ norm, but the accuracy is not satisfactory. To further improve accuracy, the authors in [25] transformed the original localization problem into a generalized trust-region subproblem (GTRS) and developed a novel weighted least squares (NWLS) with a known transmit power scheme (NWLS-K) and an unknown transmit power scheme (NWLS-U). Moreover, the authors in [26] presented a different way to solve the localization problem with an SD/SOCP scheme at the tremendous iteration cost to achieve high location accuracy. In [28], the authors have designed a robust, non-cooperative localization algorithm (RNLA); although it can reduce the estimated error in outliers, the signal loss resulting from the absorption was not considered. Overall, some methods like NWLS or SD/SCOP could improve the localization accuracy in a way, the performance might not be suitable for the dynamic scenario. Some methods like RNLA or LS may provide a fast and feasible solution, but the localization accuracy seems unsatisfactory. In this case, we would like to develop an exclusive method for the dynamic ocean environment with a relatively high localization accuracy for target localization. 

In the paper, a two-phase robust target localization method in OSNs under unknown transmission power is proposed. First, the original localization problem is described by taking the maximum likelihood criterion; then, it is transformed into an unknown transmitting-power alternating non-negativity-constrained least square problem (UT-ANLS) by applying some approximations. Furthermore, a method based on the interior point method (IPM) is developed to acquire the solution. However, the accuracy of the solution obtained by IPM may be far away from the accurate one. In this case, a majorization-minimization tactic (MMT) is proposed to refine the transmission power and target location simultaneously in the iteration in which the solution of IPM is as the initial guess. 

The main contributions of the paper can be summarized as follows:

(1) The considered localization problem is converted into a UT-ANLS framework by exploiting certain approximations.
(2) A two-phase optimization method, i.e., IPM-MM, is described to solve the problem, wherein the first is the IPM, and the second is the MMT.
(3) A closed-form expression of the Cramer–Rao lower bound (CRLB) of the measurement model is derived under the unknown transmission power.

The rest of the paper is arranged as follows. The problem is formulated in Section 2. In Section 3, the proposed method, IPM-MM, is illustrated. Numerical simulation results in different scenarios are shown and discussed in Section 4. In the last section, we make a summary of the paper.

## 2. Problem Formulation

Consider a three-dimensional (3-D) OSN with plenty of buoy nodes deployed on the ocean surface including *M* anchor nodes and an unknown node. Without loss of generality, the *i*th anchor can be denoted as $a_{i}={\left[a_{i1},a_{i2},a_{i3}\right]}^{T}$, where i=1,2,…,M. The unknown target node can be represented as $u={\left[u_{1},u_{2},u_{3}\right]}^{T}$, where *T* stands for the transpose operation. We suppose that the RSS information propagates between the *i*th anchor node and target node following the shadowing model, which could be modeled as [24,26,29]:

(1) $$P_{si}=P_{0}-10\alpha \log_{10}\frac{\left\|u-a_{i}\right\|}{d_{0}}-\alpha_{f}\left\|u-a_{i}\right\|+n_{i},$$

where $P_{si}$ is the signal strength received through the *i*th anchor node from the target node, $d_{0}^{}\left(d_{0}^{}\leq \left\|u-a_{i}\right\|\right)$ is the reference distance and it is usually set as 1 m, $P_{0}$ denotes the transmit power of the target when the distance is $d_{0}^{}$, α is the path loss exponent under different environments, ‖·‖ is $l_{2}$ norm, and $n_{i}$ is the noise with zero mean and variance $\sigma_{i}^{2}$. $\alpha_{f}$ is the absorption coefficient which can be obtained from Thorp’s formula using a frequency function, denoted as [30]:

(2) $$\alpha_{f}=0.11\frac{f^{2}}{1+f^{2}}+44\frac{f^{2}}{4100+f^{2}}+2.75\times 10^{-4}f^{2}+0.003.$$

**Figure 2:**
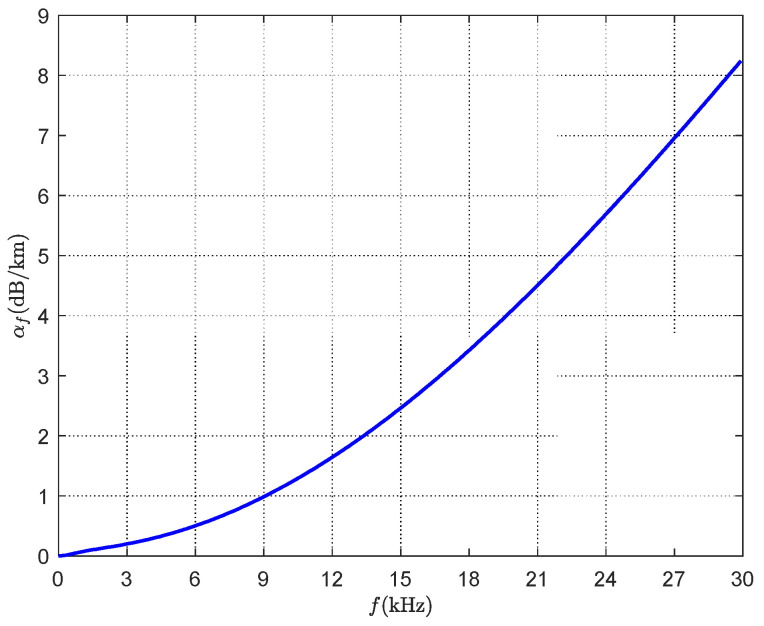
Absorption coefficient *α_f_* with variable frequency *f*.

Figure 2 shows the relationship of *α_f_* with the variable frequency *f*. According to [31,32], most acoustic systems operate in the 0–30 kHz frequency band. In addition, it deserves to be noted that a standard terrestrial localization using RSS measurements can be obtained while *α_f_* equals to 0.

Let $z_{i}=\alpha_{f}\left\|u-a_{i}\right\|$, then Equation (1) can be written as (3).

(3) $$P_{si}=P_{0}-z_{i}-10\alpha \log_{10}\frac{\left\|u-a_{i}\right\|}{d_{0}^{}}+n_{i}.$$

Given the observation matrix $P={\left[P_{s1},P_{s2},\ldots ,P_{s}{}_{M}\right]}^{T}$, and $n_{i}$ follows a Gaussian distribution with zero-mean and variance $\sigma_{i}^{2}$, the probability density function (PDF) can be expressed as [28]:

(4) $$p(P|u,P_{0})=\prod_{i=1}^{M}\frac{1}{\sqrt{2\pi }\sigma_{i}}\exp\left\{\frac{{\left(P_{si}-P_{0}+z_{i}+10\alpha \log_{10}\frac{\left\|u-a_{i}\right\|}{d_{0}}\right)}^{2}}{2\sigma_{i}^{2}}\right\}.$$

Maximizing the joint PDF, the maximum likelihood estimator can be derived as:

(5) $$E\left(\hat{u},{\hat{P}}_{0}\right)\underset{u,P_{0}}{=\arg\min}\sum_{i=1}^{M}\frac{{\left(P_{si}-P_{0}+z_{i}+10\alpha \log_{10}\frac{\left\|u-a_{i}\right\|}{d_{0}}\right)}^{2}}{2\sigma_{i}^{2}}.$$

The above problem is difficult to solve directly due to its highly non-convexity, which motivates us to simplify it using an approximation method to find the sub-optimal solution.

## 3. Proposed Method

### 3.1. UT-ANLS Framework

First, we conduct a transformation from (1) as [20,33,34]:

(6) $$\left\|u-a_{i}\right\|\approx d_{0}10^{\frac{P_{0}-P_{si}-z_{i}+n_{i}}{10\alpha }},$$

Assume $\gamma_{i}=\frac{P_{0}-P_{si}-z_{i}}{10\alpha }$, then when the noise is relatively small Equation (6) can be rewritten as:

(7) $$\left\|u-a_{i}\right\|\approx d_{0}10^{\gamma_{i}}.$$

Then, the estimation problem in Equation (5) is converted to:

(8) $$\underset{x}{\arg\min}\sum_{i=1}^{M}{\left(\left\|u-a_{i}\right\|-d_{0}10^{\gamma_{i}}\right)}^{2}.$$

We square Equation (8) and expand each term, then:

(9) $$\underset{x}{\arg\min}\sum_{i=1}^{M}{\left(\kappa -2a_{i}{}^{T}u+{\left\|a_{i}\right\|}^{2}-d_{0}{}^{2}10^{2\gamma_{i}}\right)}^{2}.$$

where $\kappa ={\left\|u\right\|}^{2}$.

Let $y={\left[u^{T},10^{\frac{P_{0}}{5\alpha }},\kappa \right]}^{T}$ be the estimation vector, the problem in (9) is finally transformed into an equivalent ANLS problem under UT in (10):

(10) $$F\left(y\right)=\underset{y\geq 0}{\arg\min}{\left\|Ay-B\right\|}^{2},$$

where:

(11) $$A={\left[\begin{array}{ccc} 2a_{1}^{T} & d_{0}{}^{2}10^{-\frac{P_{s1}+z_{i}}{5\alpha }} & -1\\ \vdots & \vdots & \vdots \\ 2a_{M}^{T} & d_{0}{}^{2}10^{-\frac{P_{sM}+z_{i}}{5\alpha }} & -1 \end{array}\right]}^{T},B=\left[\begin{array}{c} {\left\|a_{1}\right\|}^{2}\\ \vdots \\ {\left\|a_{M}\right\|}^{2} \end{array}\right].$$

The problem can be further changed to minimize a quadratic objective function under the positive constraint. 

### 3.2. PHASE1: Interior Point Method (IPM)

IPM [35] is numerically an effective solution for convex quadratic programs, which can obtain a feasible possible solution for the UT-ANLS framework. A finite number of iterations are conducted to achieve an ideal value. In this section, we introduce the IPM to solve the target localization problem based on the UT-ANLS framework.

According to IPM, we first redescribe the formula (10) by one logarithmic barrier function to transform the original problem into an unconstrained one:

(12) $$\phi \left(y,\mu \right)=\min\left({\left\|Ay-B\right\|}^{2}-\mu \sum_{i=1}^{M}\ln\left(y_{i}\right)\right),$$

where μ is a penalty factor (μ>0) and the feasible region is denoted by $D=\left\{y_{i}>0,i=1,2,\ldots ,M\right\}$.

Suppose that $f\left(y\right)={\left\|Ay-B\right\|}^{2}$, $\bar{I}\left(y\right)=-\mu \sum_{i=1}^{M}\ln\left(y\right)$, when y>0, $\bar{I}\left(y\right)$ is bounded; otherwise, it tends to be infinite, which results in ϕ(y) being infinite.

We get the estimated value through multiple iterations, and the barrier parameter of the $k^{th}$ iteration can be expressed as *μ_k_*. The above formula can be rewritten as:

(13) $$\min\phi \left(y,\mu_{k}\right)=f\left(y\right)-\mu_{k}\bar{I}\left(y\right),$$

The first-order and second-order partial derivative can be denoted as $\nabla \phi_{y}\left(y,\mu_{k}\right)=\nabla \left(f\left(y\right)\right)-\mu_{k}\cdot Y^{-1}\cdot e$, $\nabla \phi_{yy}^{2}\left(y,\mu_{k}\right)=\nabla \left(f\left(y\right)\right)-\mu_{k}\cdot Y^{-2}\cdot e$, where $Y=\mathrm{diag}\left(y_{1},y_{2},\ldots ,y_{M}\right)$, $e={\left(1,1,\ldots ,1\right)}^{T}$(the number is *M*), then the Newton-Raphson method is adopted to search the KKT solution.

Let $z_{k}={\left(y_{k}\right)}^{-1}$, $Z=\mathrm{diag}\left(z_{1},z_{2},\ldots ,z_{M}\right)$, the KKT condition can be:

(14) $$\begin{array}{r} y^{T}\left(A^{T}Ay-A^{T}b\right)-z=0,\\ YZe-\mu e=0, \end{array}$$

Then, the problem can be solved by:

(15) $$\left[\begin{array}{cc} W_{k} & -I\\ Z_{k} & Y_{k} \end{array}\right]\left[\begin{array}{c} d_{k}^{y}\\ d_{k}^{z} \end{array}\right]=-\left[\begin{array}{c} \nabla f\left(y_{k}\right)-z_{k}\\ Y_{k}Z_{k}e-\mu_{j}e \end{array}\right],$$

where $W_{k}=\nabla \phi_{yy}^{2}\left(y_{k},\mu_{k}\right)$, $d_{k}^{y}$ and $d_{k}^{z}$ are the Newton step. 

Once an initial point that satisfies the above system is found, a line search could be carried out along $d_{k}^{y}$ to find a new iteration value, following $y_{k+1}=y_{k}+\alpha_{k}\cdot d_{k}^{y}$, where $\alpha_{k}$ is the step length. Similarly, the update of z satisfies $z_{k+1}=z_{k}+\alpha_{k}\cdot d_{k}^{z}$. When the tolerance of all conditions holds, the final solution $y^{*}$ can be obtained. The whole process of IPM is shown in Algorithm 1.
**Algorithm 1** The process of IPM1. **Initiation:** initial point $y_{0}\in D_{0}$, $z_{0}$, stopping tolerance ε, $\mu_{1}>0$,    ϖ∈(0,1), k:=12. **While** ($\left|\nabla f\left(y_{k}\right)-z_{k}\right|>\varepsilon \&\&\left|Y_{k}Z_{k}e-\mu_{k}e\right|>\varepsilon$) **do**3.  Figure out the iteration solution $y_{k}$, $z_{k}$ to (15)4.  $\mu_{k+1}:=\varpi \mu_{k}$5.  k:=k+16. **End**7. final solution $y^{*}\approx y_{k}$.

Although the interior point method has a relatively low memory consumption, the accuracy of its solution may be unsatisfactory because the barrier function prevents the iteration from approaching the boundary of the inequality constraint.

### 3.3. PHASE2: Majorization-Minimization Tactic (MMT)

**Figure 3:**
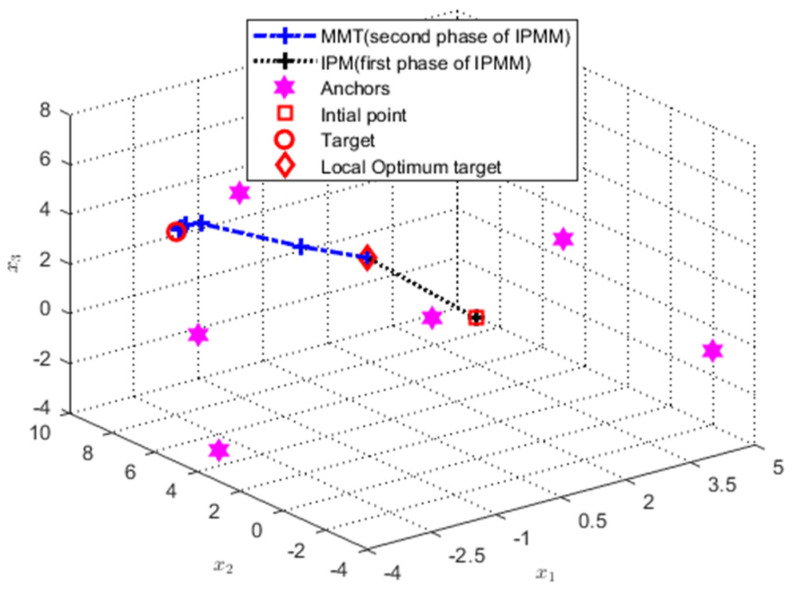
Optimal path analysis where the true target location is $u={\left[-3,7,4\right]}^{T}$, the initial point is ${\left[1,1,1\right]}^{T}$, and the anchors are $a_{1}={\left[-2,8,-1\right]}^{T}$, $a_{2}={\left[5,-2,-1\right]}^{T}$, $a_{3}={\left[-3,5,-4\right]}^{T}$, $a_{4}={\left[4,3,2\right]}^{T}$, $a_{5}={\left[-4,2,8\right]}^{T}$, and $a_{6}={\left[-2,-3,4\right]}^{T}$, respectively.

For IPM, the performance is good only when it satisfies the given constraint, once falling on the edge of restriction or out the range, it may directly tend to be an approximate local optimum solution or no solution. To acquire the best estimation, an iteration strategy, i.e., MMT, is used to optimize the solution further using the initial point computed by IPM. The whole process can be seen in Figure 3.

According to [36], we redescribe the problem (6) to construct a surrogate function:

(16) $$Q\left(u,P_{0}\right)=\underset{g\left(u\right)}{\underset{︸}{\sum_{i=1}^{M}{\left\|u-a_{i}\right\|}^{2}}}+\underset{h\left(u\right)}{\underset{︸}{\left(-2\sum_{i=1}^{M}d_{0}10^{\gamma_{i}}\left\|u-a_{i}\right\|\right)}},$$

where g(u) is obviously a convex function, and h(u) is concave.

Geometrically, the concave function h(u) can have an upper bound by using a linear function. Then, $Q\left(u,P_{0}\right)$ is made up of g(u) and the linear term. To acquire the linear term, first order Taylor expansion [37] is used for h(u) at $u^{\left(d\right)}$ where the iteration number is d.

(17) $$\begin{array}{l} h\left(u\right)\leq h\left(u^{\left(d\right)}\right)+\nabla h{\left(u^{\left(d\right)}\right)}^{T}\left(u-u^{\left(d\right)}\right)\\ =-2\sum_{i=1}^{M}d_{0}10^{\gamma_{i}}\left\|u^{\left(d\right)}-a_{i}\right\|-2\sum_{i=1}^{M}\frac{d_{0}10^{\gamma_{i}}{\left(u^{\left(d\right)}-a_{i}\right)}^{T}}{\left\|u^{\left(d\right)}-a_{i}\right\|}\left(u-u^{\left(d\right)}\right). \end{array}$$

Therefore, we denote $Q\left(u|u^{\left(d\right)},P_{0}{}^{\left(d\right)}\right)$ by:

(18) $$Q\left(u|u^{\left(d\right)},P_{0}{}^{\left(d\right)}\right)=\sum_{i=1}^{M}\left({\left\|u-a_{i}\right\|}^{2}-2d_{0}10^{\gamma_{i}}\left\|u^{\left(d\right)}-a_{i}\right\|\right)-2\sum_{i=1}^{M}\frac{d_{0}10^{\gamma_{i}}{\left(u^{\left(d\right)}-a_{i}\right)}^{T}}{\left\|u^{\left(d\right)}-a_{i}\right\|}\left(u-u^{\left(d\right)}\right).$$

Then, $P_{0}{}^{\left(d\right)}$ is fixed as $P_{0}$ using IPM, the $u^{\left(d+1\right)}$ can be updated as:

(19) $$u^{\left(d+1\right)}=\underset{u}{\arg\min}Q\left(u|u^{\left(d\right)},P_{0}\right).$$

We can obtain the concrete $u^{\left(d+1\right)}$ as:

(20) $$u^{\left(d+1\right)}=\frac{1}{M}\sum_{i=1}^{M}\left(a_{i}+\frac{d_{0}10^{\gamma_{i}}{\left(u^{\left(d\right)}-a_{i}\right)}^{T}}{\left\|u^{\left(d\right)}-a_{i}\right\|}\right).$$

Then we fix u to derive $P_{0}{}^{\left(d+1\right)}$. The update rule for $P_{0}{}^{\left(d+1\right)}$ is:

(21) $$P_{0}{}^{\left(d+1\right)}=\frac{1}{M}\sum_{i=1}^{M}\left(10\alpha \log_{10}\frac{\left\|u^{\left(d+1\right)}-a_{i}\right\|}{d_{0}}+P_{si}\right).$$

Enough attention should be paid to two conditions for its convergence: (a) The function $Q\left(u,P_{0}\right)$ is bounded below; (b) the value of $Q\left(u,P_{0}\right)$ is non-increasing, i.e., $Q\left(u^{\left(d+1\right)},P_{0}{}^{\left(d+1\right)}\right)\leq Q\left(u^{\left(d\right)},P_{0}{}^{\left(d\right)}\right)$, which implies it has a convergence limit $Q^{\left(l\right)}$. To prove the two conditions (a) and (b), we describe the process in detail as follows. 

To (a): According to the formula (16), one has $Q\left(u,P_{0}\right)\geq -\sum_{i=1}^{M}d_{0}^{2}10^{2\gamma_{i}}$,which shows that it has a lower bound.

To (b): $Q\left(u,P_{0}\right)$ consists of the concave function h(u) and convex function g(u). We use $\Theta ={\left[u,P_{0}\right]}^{T}$, then $Q\left(u,P_{0}\right)$ and $h\left(u,P_{0}\right)$ can be denoted by Q(Θ) and h(Θ). To find the upper bound of Q(Θ), we take the difference between formula (16) and formula (18) at $\Theta^{\left(d\right)}$, then we can have:

(22) $$Q\left(\Theta |\Theta^{\left(d\right)}\right)-Q\left(\Theta \right)\geq Q\left(\Theta^{\left(d\right)}|\Theta^{\left(d\right)}\right)-Q\left(\Theta^{\left(d\right)}\right),$$

Because:

(23) $$Q\left(\Theta |\Theta^{\left(d\right)}\right)-Q\left(\Theta \right)=h\left(\Theta^{\left(d\right)}\right)+\nabla h{\left(\Theta^{\left(d\right)}\right)}^{T}\left(\Theta -\Theta^{\left(d\right)}\right)-h\left(\Theta \right).$$

We find that (23) is an affine function plus a convex term, which shows that $Q\left(\Theta |\Theta^{\left(d\right)}\right)-Q\left(\Theta \right)$ is convex. Then we can get $\nabla \left(Q\left(\Theta |\Theta^{\left(d\right)}\right)-Q\left(\Theta \right)\right)|{}_{\Theta =\Theta^{\left(d\right)}}=0$. We use $C^{\left(d\right)}=Q\left(\Theta^{\left(d\right)}|\Theta^{\left(d\right)}\right)-Q\left(\Theta^{\left(d\right)}\right)$, (22) can be described by $Q\left(\Theta |\Theta^{\left(d\right)}\right)-Q\left(\Theta \right)\geq C^{\left(d\right)}$, then:

(24) $$Q\left(\Theta^{\left(d+1\right)}\right)\leq Q\left(\Theta^{\left(d+1\right)}|\Theta^{\left(d\right)}\right)-C^{\left(d\right)}\leq Q\left(\Theta^{\left(d\right)}|\Theta^{\left(d\right)}\right)-C^{\left(d\right)}\leq Q\left(\Theta^{\left(d\right)}\right).$$

### 3.4. Cramer-Rao Lower Bound (CRLB) Analysis

CRLB [38] is a covariance matrix that represents a lower bound of any unbiased linear estimators. It is usually used as the benchmark for the estimators and can be defined as the trace of the inverse of the Fisher information matrix (FIM):

(25) $$CRLB=trace\left(FIM^{-1}\right)=trace{\left[\left(\frac{\partial E}{\partial y}\right)\sum^{-1}{\left(\frac{\partial E}{\partial y}\right)}^{T}\right]}^{-1},$$

where ∑ is $diag(\sigma_{{}_{1}}^{2},\ldots ,\sigma_{{}_{M}}^{2})=diag(\sigma^{2})$:

(26) $$\frac{\partial E}{\partial y_{i}}={\left[\frac{\partial E}{\partial u},\frac{\partial E}{\partial P_{0}}\right]}^{T}={\left(\vartheta \frac{u_{1}-a_{i1}}{{\left\|u-a_{i}\right\|}^{2}}-\alpha_{f}\frac{u_{1}-a_{i1}}{\left\|u-a_{i}\right\|},\ldots ,\vartheta \frac{u_{3}-a_{i3}}{{\left\|u-a_{i}\right\|}^{2}}-\alpha_{f}\frac{u_{3}-a_{i3}}{\left\|u-a_{i}\right\|},1\right)}^{T},$$

where: $\vartheta =-\frac{10\alpha }{\ln10}$.

Let:

$$Q_{1}=\left(\vartheta \frac{u_{1}-a_{i1}}{{\left\|u-a_{i}\right\|}^{2}}-\alpha_{f}\frac{u_{1}-a_{i1}}{\left\|u-a_{i}\right\|}\right),Q_{12}=\left(\vartheta \frac{u_{1}-a_{i1}}{{\left\|u-a_{i}\right\|}^{2}}-\alpha_{f}\frac{u_{1}-a_{i1}}{\left\|u-a_{i}\right\|}\right).\left(\vartheta \frac{u_{2}-a_{i2}}{{\left\|u-a_{i}\right\|}^{2}}-\alpha_{f}\frac{u_{2}-a_{i2}}{\left\|u-a_{i}\right\|}\right)$$

$$Q_{2}=\left(\vartheta \frac{u_{2}-a_{i2}}{{\left\|u-a_{i}\right\|}^{2}}-\alpha_{f}\frac{u_{2}-a_{i2}}{\left\|u-a_{i}\right\|}\right),Q_{13}=\left(\vartheta \frac{u_{1}-a_{i1}}{{\left\|u-a_{i}\right\|}^{2}}-\alpha_{f}\frac{u_{1}-a_{i1}}{\left\|u-a_{i}\right\|}\right).\left(\vartheta \frac{u_{3}-a_{i3}}{{\left\|u-a_{i}\right\|}^{2}}-\alpha_{f}\frac{u_{3}-a_{i3}}{\left\|u-a_{i}\right\|}\right)$$

(27) $$Q_{3}=\left(\vartheta \frac{u_{3}-a_{i3}}{{\left\|u-a_{i}\right\|}^{2}}-\alpha_{f}\frac{u_{3}-a_{i3}}{\left\|u-a_{i}\right\|}\right),Q_{23}=\left(\vartheta \frac{u_{2}-a_{i2}}{{\left\|u-a_{i}\right\|}^{2}}-\alpha_{f}\frac{u_{2}-a_{i2}}{\left\|u-a_{i}\right\|}\right).\left(\vartheta \frac{u_{3}-a_{i3}}{{\left\|u-a_{i}\right\|}^{2}}-\alpha_{f}\frac{u_{3}-a_{i3}}{\left\|u-a_{i}\right\|}\right)$$

Then, the FIM can be denoted as:

(28) $$FIM=\sum^{-1}\cdot \sum_{i=1}^{M}\left(\begin{array}{cccc} Q_{1}{}^{2} & Q_{12} & Q_{13} & Q_{1}\\ Q_{12} & Q_{2}{}^{2} & Q_{23} & Q_{2}\\ Q_{13} & Q_{23} & Q_{3}{}^{2} & Q_{3}\\ Q_{1} & Q_{2} & Q_{3} & 1 \end{array}\right).$$

Suppose that $\left\|{\hat{y}}_{i}-y_{i}\right\|=er$, the root mean square error (RMSE) can be related to CRLB by:

(29) $$\sqrt{E\left(er^{2}\right)}\geq \sqrt{trace\left(FIM^{-1}\right)}≜\sqrt{CRLB}.$$

## 4. Numerical Simulation Results

In this section, several numerical experiments are conducted in different scenarios to evaluate the performance of IPM-MM. To verify the effectiveness of our proposed method, we compare it with LS [19], SRWLS [20], ASM [33], RNLA [28], and CRLB in MATLAB under 3-Dimension. Due to the ocean environment dynamics, we assume that the target and anchor nodes’ positions are not fixed at a cube area with side length Sel in each Monte Carlo trial. Unless otherwise stated, the maximum number of iterations $I_{m}$ is set to be 1000, the reference distance $d_{0}$ is 1 m, and Monte Carlo (MC) runs are 1000. The root mean square error (RMSE) is adopted to calibrate localization accuracy, which is defined as:

(30a) $$RMSE_{L}=\sqrt{\frac{1}{MC}\sum_{i=1}^{MC}{\left(\hat{x}-x\right)}^{2}},$$

(30b) $$RMSE_{P}=\sqrt{\frac{1}{MC}\sum_{i=1}^{MC}{\left({\hat{P}}_{0}-P_{0}\right)}^{2}},$$

where $\hat{x}$ and ${\hat{P}}_{0}$ are the estimates of the location x and power $P_{0}$ in the $i^{th}$ Monte Carlo trial, MC is the total number of Monte Carlo trials.

Before presenting the simulation experiment, some necessary instructions must be made. The scenarios that RMSE versus varying path loss exponent α, SNR, anchor nodes M, absorption coefficient $\alpha_{f}$, side length Sel and cumulative distribution function (CDF) of different algorithms are performed meeting the conditions of $SNR=10\cdot \log_{10}\left(\left\|x-a_{i}\right\|/\left(M\sigma^{2}\right)\right)$, whose details can be found in [20,33]. It is worth noting that the environmental noise in the ocean mainly includes the noise generated by factors such as tides and sea static pressure, seismic disturbance, boats and sea waves, and they usually obey a Gaussian distribution. Therefore, we use Gaussian noise to simulate the ocean noise. However, the influence of noise on localization cannot be seen intuitively from the previous simulation experiments. Hence, we supplement the experimental scenario in Section 4.7, wherein the relationship between parameter SNR and noise *α*^2^ does not hold.

### 4.1. Scenario with Varying Path Loss Exponent α

**Figure 4:**
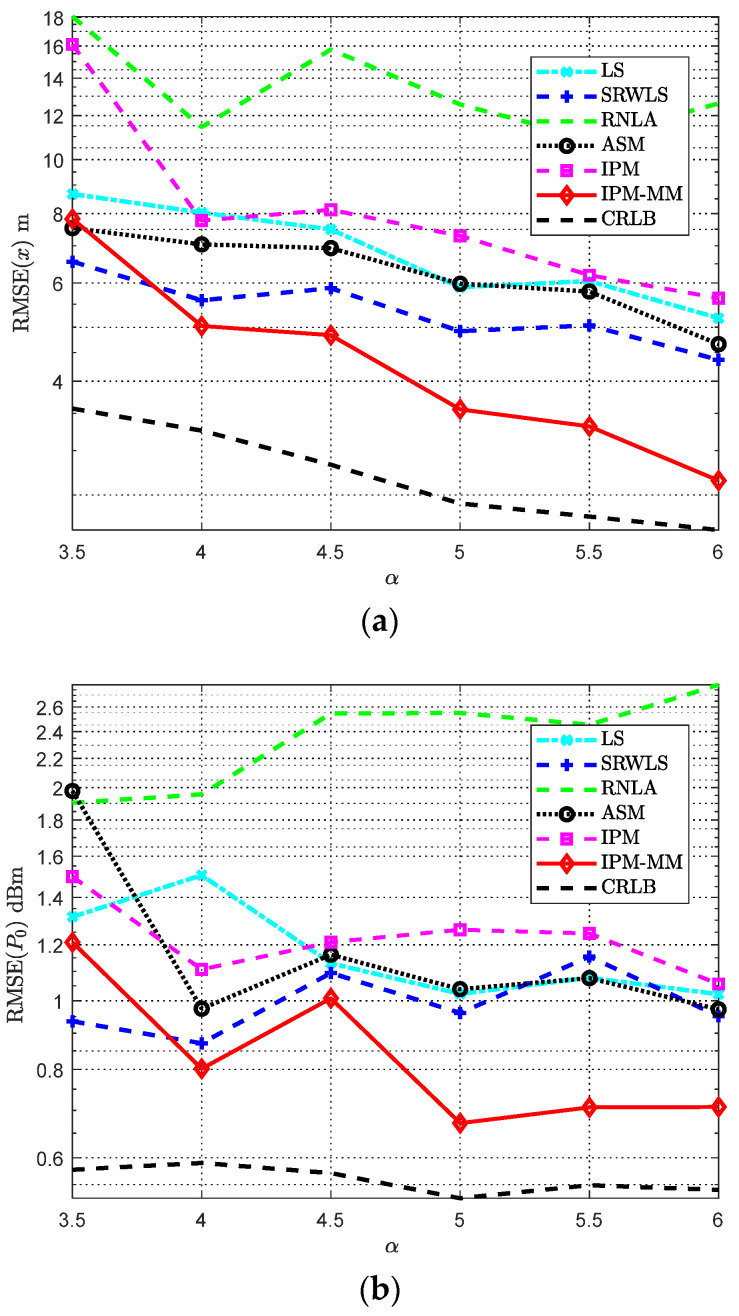
RMSE comparisons: (**a**) The RMSE of *x* versus varying *α*; (**b**) The RMSE of *P*_0_ versus varying *α*. Both with anchors *M* = 7, *α_f_* = 0.04 dB/m, *SNR* = 50 dB, and Side length *Sel* = 100 m.

The RMSE versus varying path loss exponent *α* is illustrated in Figure 4. The specific parameters are set in Table 1. It is worth mentioning that some unlisted parameters remain the same as before. Interestingly, RNLA seems more sensitive than others. It can be observed that almost all methods have acquired the relatively acceptable robustness with increasing *α* except RNLA, in which IPM-MM outperforms the others for most cases. It may be less satisfactory for the performance of the estimate of *x* and *P*_0_ when *α* = 3.5, however, with the increase of *α*, our method shows a better performance than the rest. For the single IPM, without MMT, it is less ideal compared with IPM-MM according to the simulation results, its performance is just better than RLNA when *α* ∈ [3.5, 6] shown in Figure 4a, and similarly in Figure 4b, it may not perform well among all methods, but when *α* = 4, its performance of estimating *P*_0_ is at a moderate level.

### 4.2. Scenario with Varying SNR

**Figure 5:**
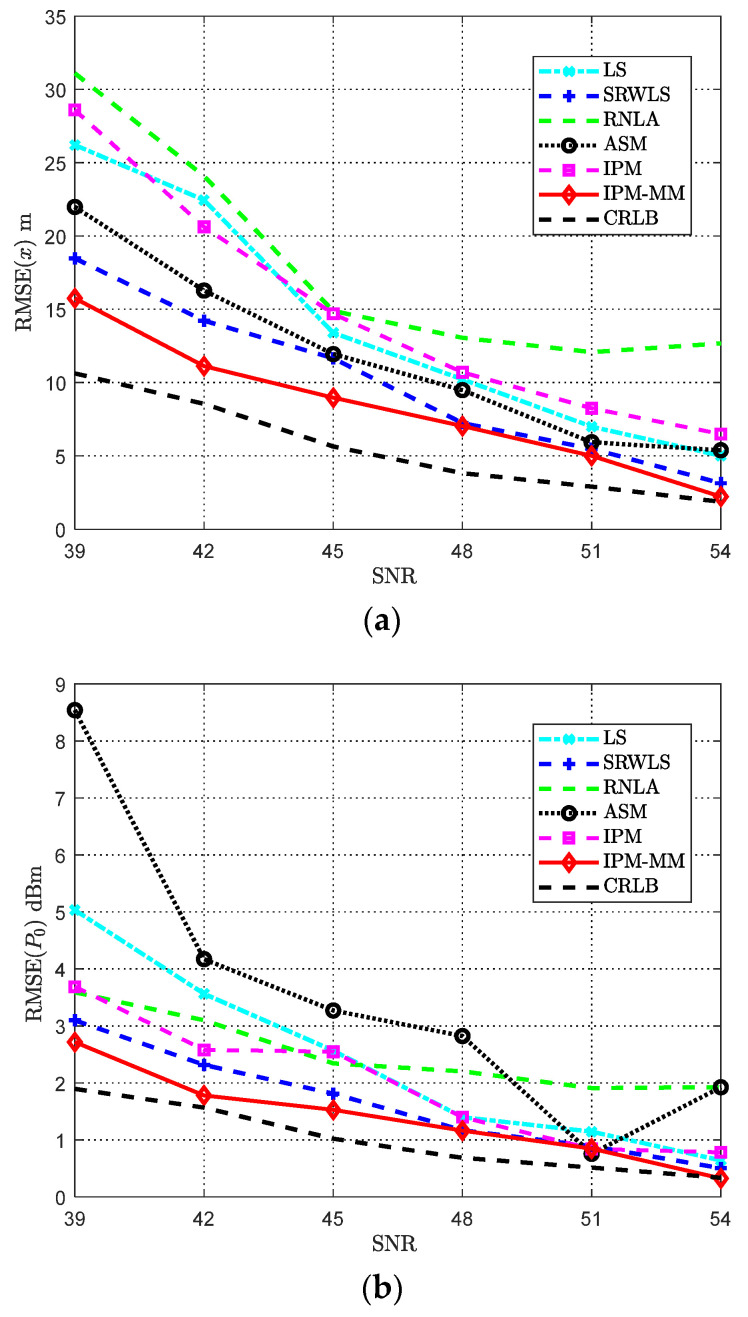
RMSE comparisons: (**a**) The RMSE of x versus varying *SNR*; (**b**) The RMSE of *P*_0_ versus varying *SNR.* Both with anchors *M* = 7, *α_f_* = 0.04 dB/m, *SNR* = 50 dB, and Side length *Sel* = 100 m.

Figure 5 depicts the RMSE of different methods with growing SNR. It is noteworthy that unless otherwise stated, all fixed parameters are the same as before. Other parameter settings are shown in Table 2. Obviously, the performance of all methods is improved when the size of *SNR* increases. Compared with LS, SRWLS, ASM, RNLA, our IPM-MM is closer to CRLB. Especially when SNR is 54 dB, our method performs better than others for both estimating *x* (shown in Figure 5a) and *P*_0_ (shown in Figure 5b). In addition, the performance gap between IPM and IPM-MM seems a little bit big, IPM-MM performs better than IPM, which demonstrates the combination is effective. Although when SNR = 51 dB, it may not evident in Figure 5b, yet it is apparent in Figure 5a. Overall, other schemes are not as good as our method in the estimation of x, and are not as close to CRLB as our scheme for estimating *P*_0_.

### 4.3. Scenario with Varying Anchor Nodes M

In this scenario, the simulation of considered methods is carried out with varying anchor nodes M. Except for some previously fixed parameters, other parameters are indicated in Table 3. The RMSE results are shown as follows. It is deserved to be pointed out that the more location information available with the increase of anchor nodes *M*. Therefore, when anchor nodes increase from 6 to 12, the performance is improved.

**Figure 6:**
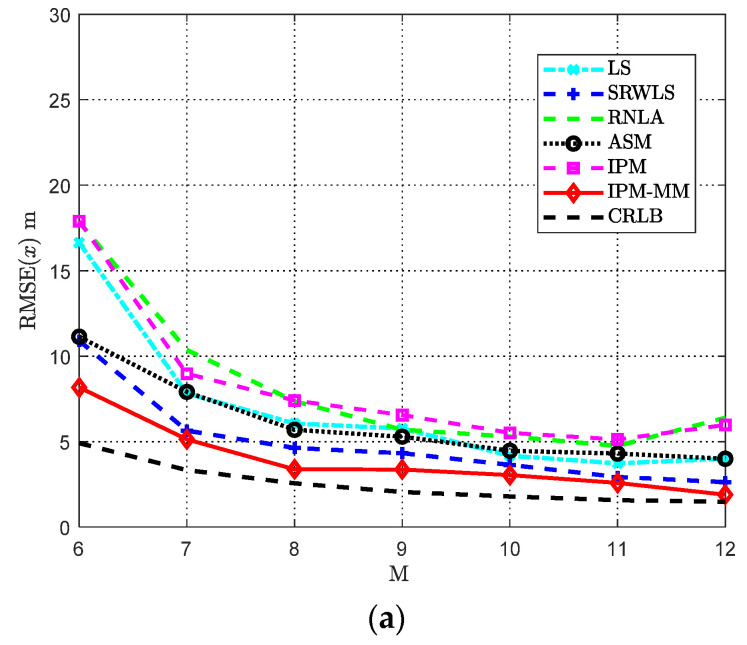

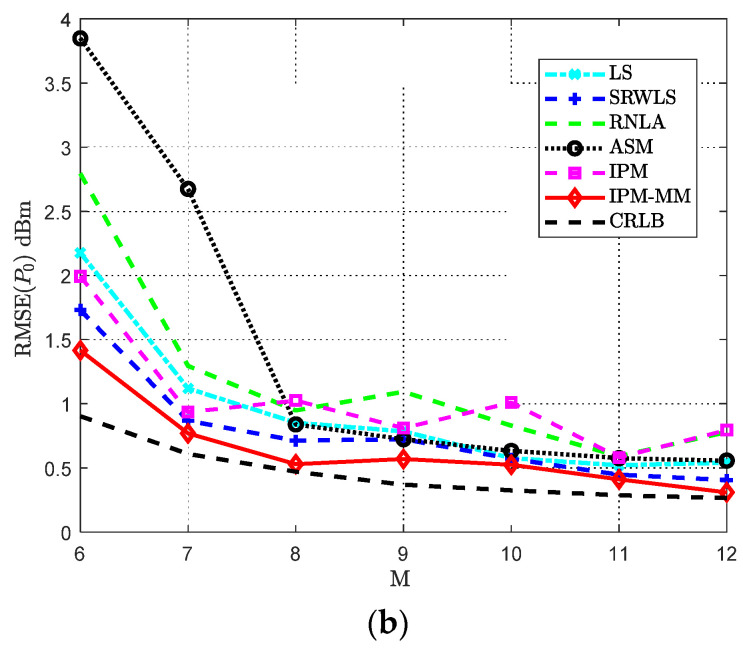
RMSE comparisons: (**a**) The RMSE of *x* versus varying *M*; (**b**) The RMSE of *P*_0_ versus varying *M*. Both with *α* = 50 dB, *α_f_* = 0.04 dB/m, *SNR* = 50 dB, and Side length *Sel* = 100 m.

From Figure 6, when the anchor’s number is on the rise, among all the methods, our proposed IPM-MM obtains a relatively satisfactory performance, in which the results are closer to the CRLB compared to others. Especially when the anchor number *M* = 8 or 12, its outperformance is remarkable. IPM may seem less robust than others under current conditions, but adding MMT surpasses the other methods and gets a perfect improvement. 

### 4.4. Scenario with Varying the Absorption Coefficient α_f_

**Figure 7:**
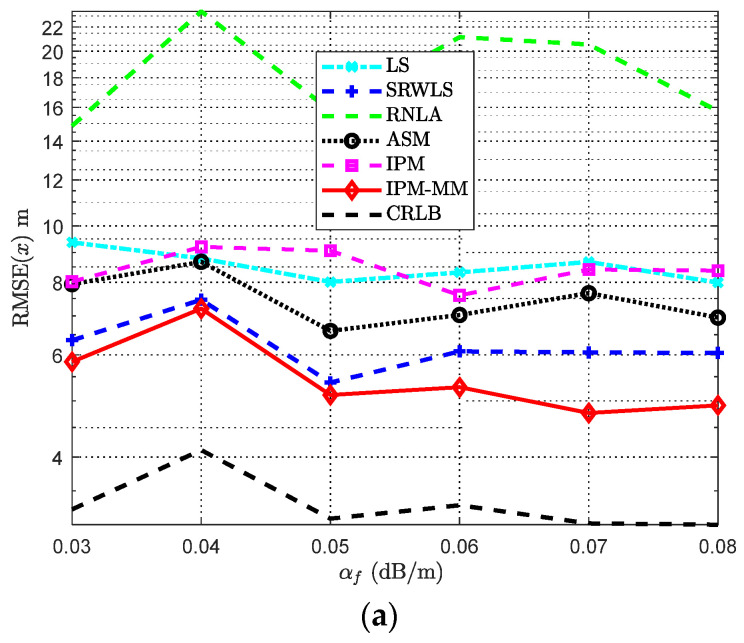

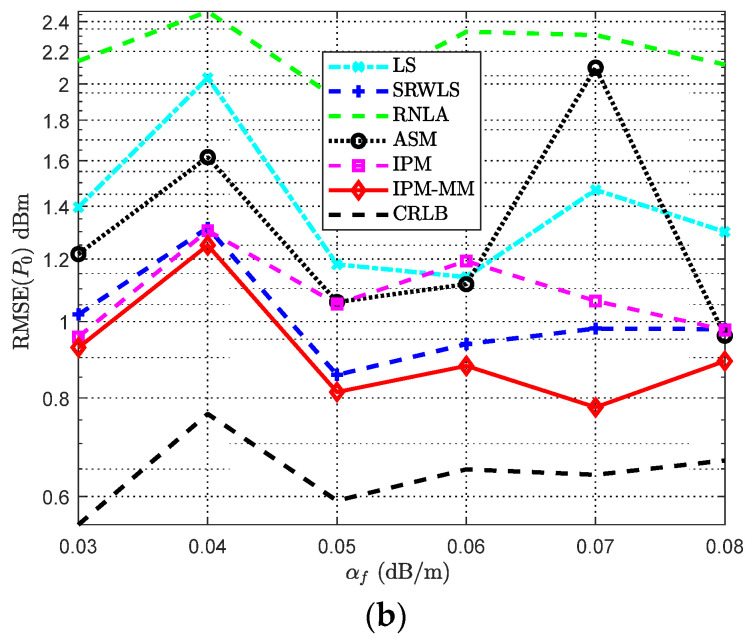
RMSE comparisons: (**a**) The RMSE of *x* versus varying *α_f_*; (**b**) The RMSE of *P*_0_ versus varying *α_f_*. Both with anchors *M* = 7, α = 4, *SNR* = 50 dB, and Side length *Sel* = 100 m.

Absorption bias plays an important role in Equation (3). It is necessary to discuss the relationship between the RMSE and absorption coefficient *α_f_*, which is displayed in Figure 7. Apart from some previously fixed parameters, other parameters are displayed in Table 4. Obviously, RNLA seems more sensitive to *α_f_* than others, and *α_f_* has a great influence on each scheme. Nevertheless, we observe that our solution acquires the relatively acceptable robustness among all the schemes and is still optimal to target location estimation. Figure 7b, in which IPM-MM outperforms the others for all cases. For the single IPM, without MMT, it is not ideal compared with IPM-MM according to the simulation results; its performance is just at a moderate level among all methods. With the increase of *α_f_*, our improvements on the basis of IPM could be more visible.

### 4.5. Scenario with Varying Side Length Sel

**Figure 8:**
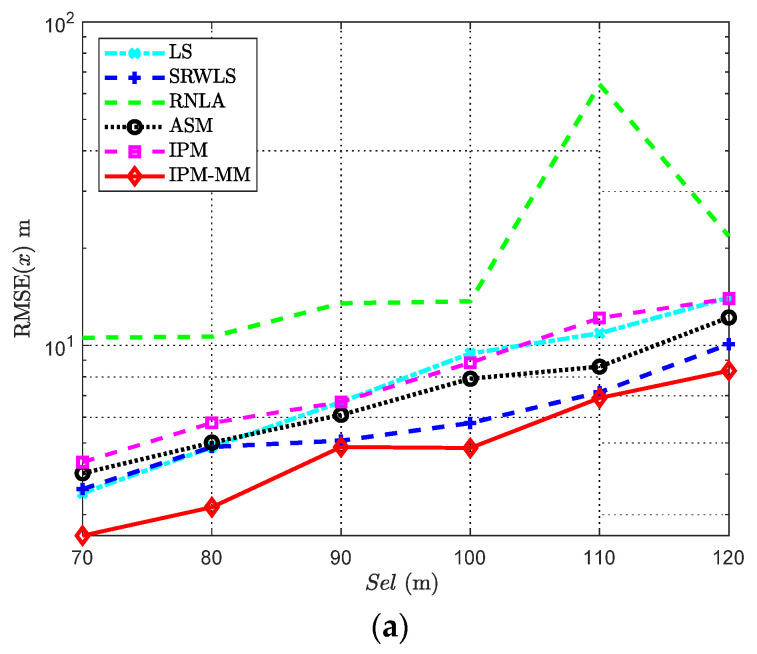

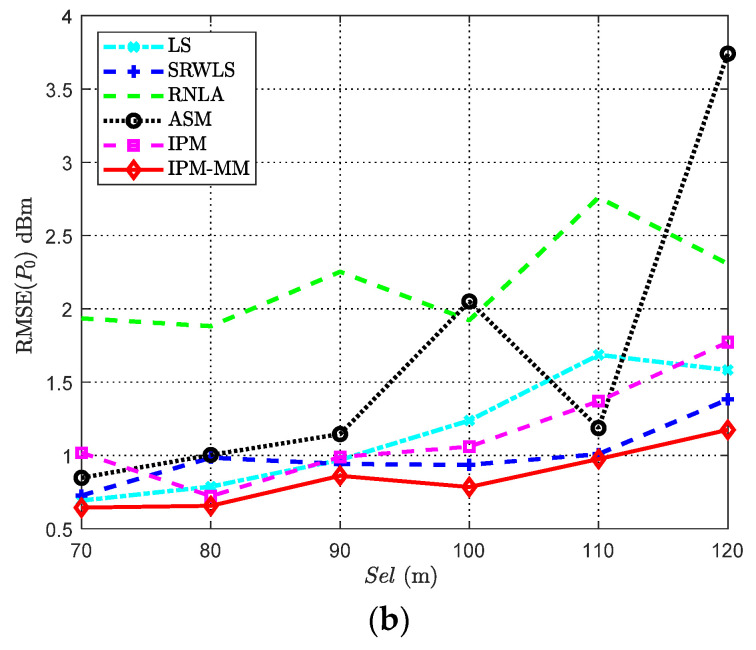
RMSE comparisons: (**a**) The RMSE of x versus varying *Sel*; (**b**) The RMSE of *P*_0_ versus varying *Sel*. Both with anchors *M* = 7, α = 4, $\alpha_{f}$ = 0.04 dB/m, and *SNR* = 50 dB.

Due to the fact that the absorption bias is determined by the absorption coefficient $\alpha_{f}$ and the distance between each anchor and the target node, it is essential to carry out the simulation experiment. The related parameters are shown in Table 5. According to Figure 8, when the side length ranges from 70 m to 120 m, except for a sensitive method RNLA, the RMSE of other algorithms is increasing because the adverse impact of the absorption bias would rise synchronously. In light of the results, IPM-MM has a relatively low error among all the considered methods. It can be found that when *Sel* equals 110 m, its performance is similar to SRWLS, but is better than other methods with side range is less than 110 m, and interestingly, when the range is up to 120 m, it recovers its good performance. It is worth noting that when *Sel* = 80 m, the IPM-MM performance of estimating $P_{0}$ is slightly close to IPM. However, IPM-MM acquires great improvements on the basis of IPM under other conditions. For ensuring location accuracy and good power estimation, our IPM-MM is effective and recommendable to some extent.

### 4.6. Cumulative Distribution Function (CDF)

**Figure 9:**
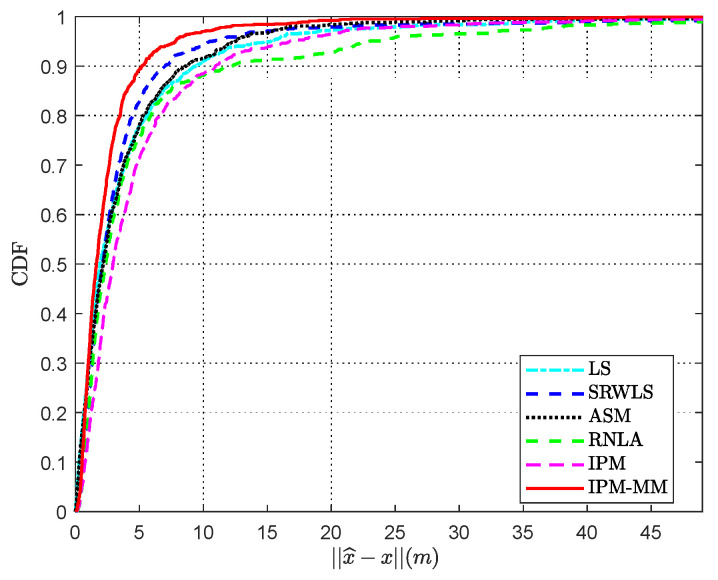
Cumulative distribution function (CDF) versus error $\left\|\hat{x}-x\right\|$ with α=4, SNR = 50 dB, *α_f_* = 0.04 dB/m, Side length *Sel* = 100 m, and 7 anchors.

Figure 9 indicates the cumulative distribution function (CDF) of various methods versus $\left\|\hat{x}-x\right\|$ when $\alpha_{f}$ = 0.04 dB/m. The other parameter settings are shown in Table 6. It is obvious that our proposed method obtains the best performance. When CDF reaches almost 90%, the error $\left\|\hat{x}-x\right\|$ of our IPM-MM is 5.20 m, whereas the errors of SRWLS, ASM, LS, IPM, RNLA are 7.05, 8.70, 9.25, 10.94 and 12.01 m, respectively. According to the numerical results, we find that IPM combined with the majorization-minimization strategy brings better improvement than the single IPM and realize 52.5% performance improvement.

### 4.7. Scenario with Varying Noise $\sigma^{2}$

**Figure 10:**
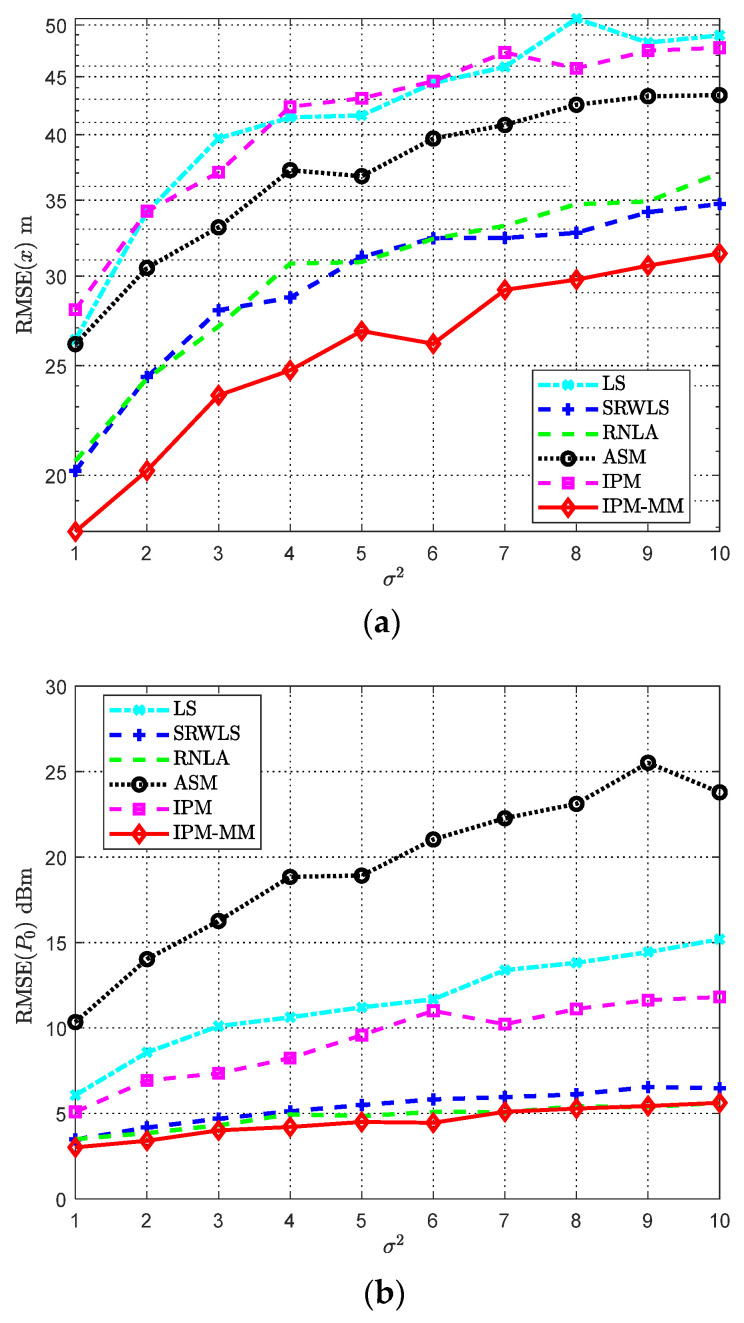
RMSE comparisons: (**a**) The RMSE of *x* versus varying $\sigma^{2}$; (**b**) The RMSE of *P*_0_ versus varying $\sigma^{2}$. Both with, α = 4, $\alpha_{f}$ = 0.04 dB/m, *Sel* = 100, and anchors *M* = 7.

Figure 10 depicts the RMSE of various methods with growing $\sigma^{2}$. The noise parameter range is set from 1 to 10. The relevant parameters are displayed in Table 7. Intuitively, with the increase of $\sigma^{2}$, the overall performance shows a downward trend. Compared with LS, SRWLS, ASM, RNLA, our IPM-MM has a lower RMSE especially estimating x. Although in Figure 10b, our proposed method has no obvious advantages compared with SRWLS and RNLA, our scheme is more accurate for the position estimation. In addition, IPM-MM always presents a better performance for the estimate of x and $P_{0}$ than IPM. Besides, we find that the performance gap between SRWLS and RNLA is small; it occurs because both two converted the location problem into a GTRS. Generally, to ensure good performance in a noisy environment, our method provides the best choice of all.

## 5. Conclusions

In this paper, a novel localization method called IPM-MM is proposed in OSNs using RSS measurements, which is applicable for the unknown transmission power case. The objective function of the original localization problem is transformed into the UT-ANLS framework, and the IPM together with the MMT is exploited to find an optimum solution. The simulation results prove that our proposed IPM-MM method can be superior to other state-of-the-art approaches in terms of localization accuracy in a relatively high dynamics environment.

## Figures and Tables

**Table 1:** Parameters in the scenario with varying path loss exponent *α*.

Parameters	Value
*α_f_* (Absorption coefficient)	0.04 dB/m
*SNR* (Signal noise ratio)	50 dB
*M* (The anchor number)	7
*Sel* (Side length)	100 m

**Table 2:** Parameters in the scenario with varying the absorption coefficient *α_f_*.

Parameters	Value
*α* (Path loss exponent)	4
*α_f_* (Absorption coefficient)	0.04 dB/m
*M* (The anchor number)	7
*Sel* (Side length)	100 m

**Table 3:** Parameters in the scenario with varying anchor nodes *M*.

Parameters	Value
*α* (Path loss exponent)	4
*α_f_* (Absorption coefficient)	0.04 dB/m
SNR (Signal noise ratio)	50 dB
Sel (Side length)	100 m

**Table 4:** Parameters in the scenario with varying the absorption coefficient *α_f_*.

Parameters	Value
α (Path loss exponent)	4
*SNR* (Signal noise ratio)	50 dB
*M* (The anchor number)	7
*Sel* (Side length)	100 m

**Table 5:** Parameters in the scenario with varying side length *Sel*.

Parameters	Value
α (Path loss exponent)	4
*α_f_* (Absorption coefficient)	0.04 dB/m
*SNR* (Signal noise ratio)	50 dB
*M* (The anchor number)	7

**Table 6:** Parameters in the scenario with varying noise $\sigma^{2}$.

Parameters	Value
α (Path loss exponent)	4
*SNR* (Signal noise ratio)	50 dB
*Sel* (Side length)	100 m
*M* (The anchor number)	7

**Table 7:** Parameters in the scenario with varying noise $\sigma^{2}$.

Parameters	Value
*α* (Path loss exponent)	4
*α_f_* (Absorption coefficient)	0.04 dB/m
*Sel* (Side length)	100 m
*M* (The anchor number)	7

## References

[1] Collotta M., Pau G., Costa D.G. (2018). A Fuzzy-based Approach for Energy-Efficient Wi-Fi Communications in Dense Wireless Multimedia Sensor Networks. Comput. Netw..

[2] Chakraborty S., Goyal N.K., Soh S. (2020). On Area Coverage Reliability of Mobile Wireless Sensor Networks With Multistate Nodes. IEEE Sens. J..

[3] Wang T., Wang X., Shi W., Zhao Z., He Z., Xia T. (2020). Target localization and tracking based on improved Bayesian enhanced least-squares algorithm in wireless sensor networks. Comput. Netw..

[4] Luo H., Wu K., Gong Y., Ni L.M. (2016). Localization for Drifting Restricted Floating Ocean Sensor Networks. IEEE Trans. Veh. Technol..

[5] Chitre M., Shahabudeen S., Stojanovic M. (2008). Underwater Acoustic Communications and Networking: Recent Advances and Future Challenges. Mar. Technol. Soc. J..

[6] Saeed N., Celik A., Al-Naffouri T.Y., Alouini M.-S. (2019). Underwater optical wireless communications, networking, and localization: A survey. Ad Hoc Netw..

[7] Zhang W., Han G., Wang X., Guizani M., Fan K., Shu L. (2020). A Node Location Algorithm Based on Node Movement Prediction in Underwater Acoustic Sensor Networks. IEEE Trans. Veh. Technol..

[8] Yun C., Choi S. (2020). A Feasibility Analysis of an Application-Based Partial Initialization (API) Protocol for Underwater Wireless Acoustic Sensor Networks. Sensors.

[9] Mohammadi Z., Soleimanpour-Moghadam M., Askarizadeh M., Talebi S. (2020). Increasing the Lifetime of Underwater Acoustic Sensor Networks: Difference Convex Approach. IEEE Syst. J..

[10] Han S., Li L., Li X. (2020). Deep Q-Network-Based Cooperative Transmission Joint Strategy Optimization Algorithm for Energy Harvesting-Powered Underwater Acoustic Sensor Networks. Sensors.

[11] Wu H., Mei X., Chen X., Li J., Wang J., Mohapatra P. (2018). A novel cooperative localization algorithm using enhanced particle filter technique in maritime search and rescue wireless sensor network. ISA Trans..

[12] Sadeghi M., Behnia F., Amiri R. (2020). Optimal Sensor Placement for 2-D Range-only Target Localization in Constrained Sensor Geometry. IEEE Trans. Signal Process..

[13] Mei X., Wu H., Xian J., Chen B. (2021). RSS-Based Byzantine Fault-Tolerant Localization Algorithm Under NLOS Environment. IEEE Commun. Lett..

[14] Ketabalian H., Biguesh M., Sheikhi A. (2020). A Closed-Form Solution for Localization Based on RSS. Trans. Aerosp. Electron. Syst..

[15] Prasad K.N.R.S.V., Bhargava V.K. (2021). RSS Localization Under Gaussian Distributed Path Loss Exponent Model. IEEE Wirel. Commun. Lett..

[16] Achroufene A., Amirat Y., Chibani A. (2019). RSS-Based Indoor Localization Using Belief Function Theory. IEEE Trans. Autom. Sci. Eng..

[17] Wang G., Chen H., Li Y., Jin M. (2012). On Received-Signal-Strength Based Localization with Unknown Transmit Power and Path Loss Exponent. IEEE Wirel. Commun. Lett..

[18] Lohrasbipeydeh H., Gulliver T.A., Amindavar H. (2015). Unknown Transmit Power RSSD Based Source Localization with Sensor Position Uncertainty. IEEE Trans. Commun..

[19] Yu K. (2007). 3-D localization error analysis in wireless networks. IEEE Trans. Wirel. Commun..

[20] Tomic S., Beko M., Dinis R. (2016). 3-D target localization in wireless sensor networks using RSS and AoA measurements. IEEE Trans. Veh. Technol..

[21] Gädeke T., Schmid J., Krüger M., Jany J., Stork W., Müller-Glaser K.D. A bi-modal ad-hoc localization scheme for wireless networks based on RSS and ToF fusion. Proceedings of the 2013 10th Workshop on Positioning, Navigation and Communication (WPNC).

[22] Chan Y.T., Chan F., Read W., Jackson B.R., Lee B.H. Hybrid localization of an emitter by combining angle-of-arrival and received signal strength measurements. Proceedings of the 2014 IEEE 27th Canadian Conference on Electrical and Computer Engineering (CCECE).

[23] Tomic S., Beko M., Tuba M. (2019). A Linear Estimator for Network Localization Using Integrated RSS and AOA Measurements. IEEE Signal Process. Lett..

[24] Xu T., Hu Y., Zhang B., Leus G. RSS-based sensor localization in underwater acoustic sensor networks. Proceedings of the ICASSP2016.

[25] Chang S., Li Y., He Y., Hui W. (2018). Target Localization in Underwater Acoustic Sensor Networks Using RSS Measurements. Appl. Sci..

[26] Chang S., Li Y., He Y., Wu Y. (2019). RSS-Based Target Localization in Underwater Acoustic Sensor Networks via Convex Relaxation. Sensors.

[27] Saeed N., Celik A., Al-Naffouri T.Y., Alouini M.-S. (2018). Energy Harvesting Hybrid Acoustic-Optical Underwater Wireless Sensor Networks Localization. Sensors.

[28] Mei X., Wu H., Xian J., Chen B., Zhang H., Liu X. (2019). A robust, non-cooperative localization algorithm in the presence of outlier measurements in ocean sensor networks. Sensors.

[29] Zhang B., Wang H., Xu T., Zheng L., Yang Q. Received signal strength-based underwater acoustic localization considering stratification effect. Proceedings of the IEEE Oceans 2016.

[30] Qarabaqi P., Stojanovic M. (2013). Statistical characterization and computationally efficient modeling of a class of underwater acoustic communication channels. IEEE J. Ocean. Eng..

[31] Cui J.H., Kong J., Gerla M., Zhou S. (2006). The challenges of building mobile underwater wireless networks for aquatic applications. IEEE Netw..

[32] Stojanovic M., Preisig J. (2009). Underwater acoustic communication channels: Propagation models and statistical characterization. IEEE Commun. Mag..

[33] Mei X., Wu H., Xian J. (2020). Matrix Factorization-Based Target Localization via Range Measurements with Uncertainty in Transmit Power. IEEE Wirel. Commun. Lett..

[34] Mei X., Wu H., Saeed N., Ma T., Xian J., Chen Y. (2020). An Absorption Mitigation Technique for Received Signal Strength-Based Target Localization in Underwater Wireless Sensor Networks. Sensors..

[35] Sousa A.A., Torres G.L., Canizares C.A. (2011). Robust Optimal Power Flow Solution Using Trust Region and Interior-Point Methods. IEEE Trans. Power Syst..

[36] Sun Y., Babu P., Palomar D.P. (2017). Majorization minimization algorithms in signal processing, communications, and machine learning. IEEE Trans. Signal Process..

[37] Beck A. (2017). First-Order Methods in Optimization.

[38] Steven M.K. (1993). Fundamentals of statistical signal processing. Technometrics.

